# On the Non‐Catalytic Role of Lytic Polysaccharide Monooxygenases in Boosting the Action of PETases on PET Polymers

**DOI:** 10.1002/cssc.202401350

**Published:** 2024-11-14

**Authors:** Thamy L. R. Corrêa, Ellen K. B. Román, Carlos A. R. Costa, Lucia D. Wolf, Richard Landers, Peter Biely, Mario T. Murakami, Paul H. Walton

**Affiliations:** ^1^ Department of Chemistry University of York Heslington York YO10 5DD United Kingdom; ^2^ Molecular and Morphofunctional Biology Graduate Program Institute of Biology University of Campinas (UNICAMP) Campinas Brazil; ^3^ Brazilian Nanotechnology National Laboratory (LNNano) Brazilian Center for Research in Energy and Materials (CNPEM) Campinas Brazil; ^4^ Brazilian Biorenewables National Laboratory (LNBR) Brazilian Center for Research in Energy and Materials (CNPEM) Campinas Brazil; ^5^ Institute of Physics Gleb Wataghin University of Campinas (UNICAMP) Campinas Brazil; ^6^ Institute of Chemistry Slovak Academy of Sciences Bratislava Slovak Republic

**Keywords:** plastics, pet, LPMO, PETase, bioeconomy

## Abstract

Synthetic polymers are resistant to biological attack, resulting in their long‐term accumulation in landfills and in natural aquatic and terrestrial habitats. Lytic polysaccharide monooxygenases (LPMOs) are enzymes which oxidatively cleave the polysaccharide chains in recalcitrant polysaccharides such as cellulose. It has been widely hypothesised that LPMOs could be used to aid in the enzymatic breakdown of synthetic polymers. Herein, through the use of biochemical assays, X‐ray photoelectron spectroscopy (XPS) and atomic force microscopy (AFM) we show that LPMOs can bind to polyethylene terephthalate (PET), and – in doing so – the hydrophobic surface of PET becomes more hydrophilic such that product release is boosted by subsequent treatment with classical PETases. The boosting effect is however, only observed in reactions when the LPMO and the PETase are added sequentially rather than simultaneously to the PET. Moreover, the same boosting effect is also seen when a catalytically‐inactive mutant of LPMO is used, showing that the principal means by which AA9 LPMOs boost the degradation of synthetic polymers is through their role as a “hydrophobin” rather than as an oxygenase. Indeed, in accord with this role of LPMOs, we further show that this effect can be extended to other ostensibly ‘non‐catalytic’ proteins beyond LPMOs, such as bovine serum albumin and lactate dehydrogenase.

## Graphical abstract

Using a combination of microscopy and spectroscopy techniques, we show that the pre‐treatment of PET with LPMO boosts the activity of PETase through a non‐catalytic mechanism akin to that observed for hydrophobins. This effect can be extended to other ostensibly ‘non‐catalytic’ proteins beyond LPMOs, such as bovine serum albumin and lactate dehydrogenase.

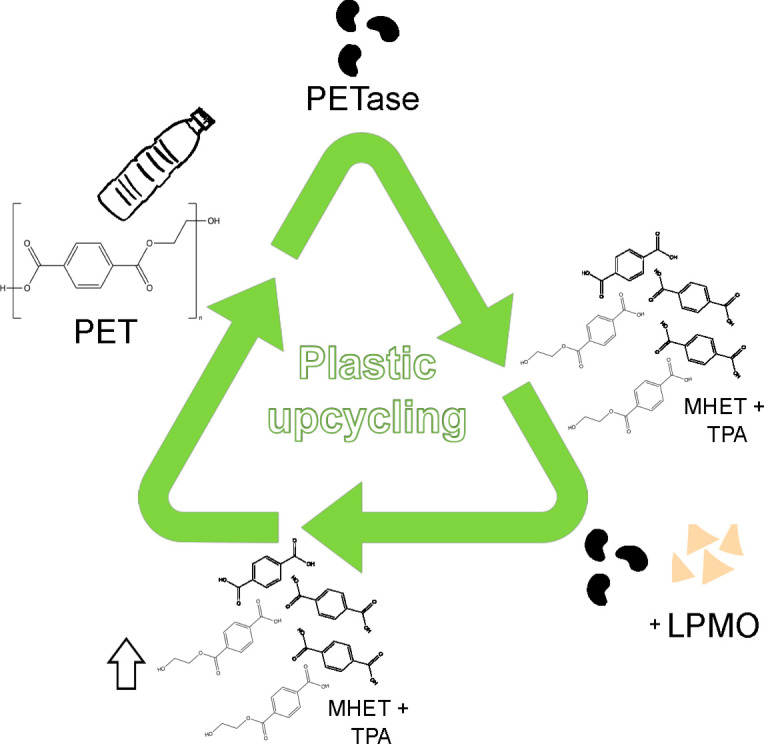

## Introduction

The mechanical and chemical properties of plastics, such as strength and inertness, underpin their global use across a myriad of applications. Accordingly, synthetic plastics are produced at scale, and at the current rate it is estimated that the total production of plastics will have exceeded 0.5 billion metric tons by 2050.^[1]^ Once used and discarded however, the very same properties of inertness and mechanical strength, combined with ineffectual recycling initiatives, have led to accumulation in landfills and in natural environments.[^1^, ^2^, ^3^, ^4^] The unchecked build‐up of plastic waste is now an existential problem for many lifeforms. For instance, the increase in microplastics in oceanic pelagic zones is an emerging environmental catastrophe, due to the toxicity of these materials to nearly all levels of life.[^5^, ^6^, ^7^, ^8^]

Notwithstanding the major issues created by waste plastic accumulation, organisms have adapted to their presence. Some bacteria, for example the polyethylene terephthalate (PET)‐assimilating bacterium *Ideonella sakaiensis*, have evolved metabolic capabilities to exploit these materials as an energy source.^[9]^ In this case, the bacterium secretes an esterase enzyme (PETase) onto the PET which then breaks down the polymeric chains through hydrolysis of the ester groups, affording mono(2‐hydroxyethyl)‐terephthalate (MHET), terephthalic acid (TPA), and minor amounts of bis(2‐hydroxyethyl)‐terephthalate (BHET).[^9^, ^10^, ^11^, ^12^, ^13^]

In the same vein, substrate inertness and hydrophobicity are faced by organisms (*e. g*. fungi and bacteria) which utilise natural biomass like cellulose.^[14]^ In fact, recognising the potential of these organisms to valorise biomass, much research effort has been devoted to investigating the enzymes used to effect the cleavage of the polysaccharide chains, with the aim of using them to release the calorific and chemical potential of constituent monomers for onward use, *e. g*. in the production of second‐generation bioethanol.^[15]^ From this research effort, a consensus model of enzyme action on polysaccharides has emerged, in which multiple different enzymes act in concert to degrade the substrate, highlighting the important synergistic effects which occur when using enzymes with different modes of action.^[16]^ In particular, LPMO enzymes, discovered in 2010/11, have a flat surface rich in aromatic residues that favours interaction with the crystalline patches of polysaccharides, which are otherwise inaccessible to canonical hydrolases. These enzymes utilize a powerful oxidative mechanism to cleave polysaccharide chains.^17–19^

Given the broad parallels between the properties of PET and polysaccharides, we hypothesized whether LPMOs could boost the action of PETases on PET through a similar oxidative cleavage mechanism, thus helping to overcome the recalcitrance of the PET polymer. In forming this hypothesis, we were encouraged by the fact that dual enzyme treatment of PET with PETases and another enzyme is known to enhance PET degradation to some extent.^[20]^ Additionally, and germane to this work, a previous study of an LPMO acting in concert with PETase exhibited enhanced activity, albeit that the details of how the LPMO caused such a boosting effect were not elucidated.^[21]^ We demonstrate here that the pre‐treatment of PET with an AA9 LPMO boosts the activity of PETase, but – in contrast to expectations – the boosting effect happens through a non‐catalytic mechanism akin to that observed for hydrophobins^[22]^ where the LPMO bound to the PET surface makes the hydrophobic substrate more accessible to PETase activity.

## Experimental

### Cloning and Expression of AfLPMO9A, KpLPMO10 A and IsPETases

*KpLPMO10 A* was cloned as described in.^[23]^ *AfLPMO9A* (GenBank XM_001264506.1), with *pelB* replacing its native signal peptide, was codon‐optimised for expression in *Escherichia coli*, synthesised and cloned into *Nde*I and *Xho*I restriction sites of pET‐21b(+) by Genscript (Piscataway, NJ, USA). *E. coli* Shuffle harbouring pET‐22b(+)‐*Kp*LPMO10 A or pET‐21b(+)‐*AfLPMO9A* were grown in TB medium at 30 °C/250 rpm until reaching OD_600_ 0.6. Then, isopropyl β‐D‐1‐thiogalactopyranoside (IPTG, 0.5 M) was added to the cultures and let overnight at 16 °C/180 rpm. The cells were centrifuged at 4 °C/7,500 rpm/30 min and lysed by osmotic shock method.^[24]^ *Kp*LPMO10 A and *Af*LPMO9A were purified by affinity chromatography in 5 mL His‐Trap column (GE Healthcare, Chicago, IL, USA) following a gradient of imidazole (0.02 M potassium phosphate pH 7.4, 0.5 M NaCl and 0.5 M imidazole). Fractions containing the target proteins were pooled, concentrated with 10 kDa Vivaspin (Sartorius, Goettingen, Germany) and applied onto HiLoad Superdex G‐75 16/60 column (GE Healthcare, Chicago, IL, USA). Fractions containing the proteins were pooled, concentrated, and treated with excess CuSO_4_ overnight at 4 °C. The excess of copper was removed with the desalting column Sephadex G‐25 PD‐10 (GE Healthcare, Chicago, IL, USA). *Af*LPMO9A^H1A^ was synthesised and cloned in the same vector as *Af*LPMO9A by Genscript. The purification strategy used was the same adopted for *Af*LPMO9A.

*IsPETase* (GenBank: BBYR01000074.1, excluding the signal peptide) was codon optimised, synthesised and cloned into *Nde*I and *Xho*I restriction sites of pET21b(+) by Genscript (Piscataway, NJ, USA). *Is*PETase was heterologously expressed in *E. coli* BL21(DE3) grown in 2x YT medium using the same protocol as for LPMOs. The cultures were centrifuged and lysed by the addition of lysozyme, 0.125 mg/mL (Sigma‐Aldrich, St. Louis, MO, USA) and sonication cycles (45 sec on/15 sec off; 15 min). *Is*PETase was purified, adopting the same steps as for LPMOs. Purified samples of *Kp*LPMO10 A, *Af*LPMO9A, *Af*LPMO9A^H1A^ and *Is*PETase were stored at 4 °C until use. *Kp*LPMO10 A, *Af*LPMO9A, *Af*LPMO9A^H1A^ and *Is*PETase concentrations were estimated (mg/mL) at all purification steps using a Nanodrop spectrophotometer (Thermo Scientific, San Jose, CA, USA). *IsPETase* ^*S121E/D186H/R280A*^ was cloned and purified as recommended in.^[25]^

The molecular mass (kDa) and molar extinction coefficient (M^−1^ cm^−1^) for all proteins used in this work: *Kp*LPMO10 A, 21/64400, *Af*LPMO9A, 25/46870, *Is*PETase and *Is*PETase^S121E/D186H/R280A^, 28/39420; were calculated from protein primary sequences using the ExPASy‐ProtParam Tool (https://web.expasy.org/protparam/).

### Other Enzymes Used in this Work

Bovine serum albumin (BSA) and lactate dehydrogenase (LD) were acquired from Sigma‐Aldrich (St. Louis, MO, USA). The molecular mass and molar extinction coefficient used for BSA (66 kDa/40800 M^−−1^ cm^−1^, respectively) and LD (36 kDa/41940 M^−1^ cm^−1^, respectively) were extracted from protein primary sequences using the ExPASy‐ProtParam Tool (https://web.expasy.org/protparam/).

### Enzyme Assays ‐ LPMOs

Coupons (~0.6×0.4 cm) of PET bottle (Crystal water, the Coca Cola company, 250 μm thickness), HDPE (juice bottle, Maluger), and PP (ice cream packaging, Kibon) were incubated in 0.05 M sodium phosphate buffer, pH 6.0, 1 mM ascorbate and 0.5, 2, or 4 μM *Kp*LPMO10 A or *Af*LPMO9A – with or without ascorbate (1 mM) ‐ for 48 h at 37 °C/850 rpm. Control reactions adopted in this work include substrate+buffer; substrate+buffer+ascorbate; substrate+buffer+H_2_O_2_ (100 μM); substrate+buffer+copper sulfate (CuSO_4_, 4 μM); substrate+buffer+CuSO_4_+H_2_O_2_. After incubation, the supernatants were heated at 99 °C/5 min, and kept at −20 °C until use or added with the same volume of ethyl acetate and sonicated for 20 min. This step was repeated twice and the organic fractions were pooled, dried overnight and kept at −20 °C until use. The residual PET was washed following procedures “i” or “ii” (described in the section “synergy assays”). In reactions with denatured *Af*LPMO9A, the enzyme was boiled for 5 min at 99 °C and immediately added to PET. The supernatants/their organic fractions, and residual substrates were employed for products detection or in polymer surface assays (described below), respectively. All reactions were carried out in triplicate.

Reactions of 2 μM *Af*LPMO9A with PASC (0.3 mg/mL) and different concentrations of PET (0.3, 1.0, 8.0 mg/mL) were carried out in 0.05 M sodium phosphate buffer, pH 6.0, at 37 °C/850 rpm for 16 h. Cello‐oligosaccharides were detected as in “detection of products from LPMO”. All reactions were carried out in triplicate.

### Polymer Surface Assays

#### X‐Ray Photoelectron Spectroscopy (XPS)

XPS spectra from plastic coupons were obtained with a VSW HA‐100 spherical analyser and AlKa radiation (hv=1486.6 eV). The high‐resolution spectra were measured with constant analyser pass energies of 44 eV, which produces a full width at half‐maximum (FWHM) line width of 1.6 eV for the Au (4f7/2) line. The pressure during the measurements was less than 6×10−8 mbar. The samples were fixed in a stainless steel sample holder and analysed without further preparation. Surface charging was corrected shifting all spectra to ensure that the C(1 s) line due to adventitious carbon was at 284.6 eV. Curve fitting was performed using Gaussian line shapes and the Shirley background was subtracted from the data. X‐Ray satellites were also subtracted when necessary. XPS data were acquired in triplicate.

#### Atomic Force Microscopy (AFM)

The deposition of LPMOs on plastic coupons was evaluated by AFM in a Multimode 8‐HR instrument (Bruker, Santa Barbara, CA, USA) in PeakForce tapping mode setup at room temperature and <5 % relative humidity. The topographic images were acquired by scanning 5×5 μm, 3×3 μm and 1×1 μm areas of the samples surface at 512×512 pixels with the silicon nitrides probes ScanAsyst‐Air (Bruker, Santa Barbara, CA, USA) using a nominal force constant at 0.4 N/m and tip apex diameter <12 nm. At least three images were taken for each sample. The images were processed using Gwyddion software.

### Detection of Products from LPMO

#### Matrix‐Assisted Laser Desorption/Ionisation Time‐of‐Flight Mass Spectrometry (MALDI‐ToF MS)

The residual PET coupons were dissolved in phenol:chloroform (2 : 3). One microliter of each sample was added to the same volume of α‐cyano‐4‐hydroxycinnamic acid (CHCA) matrix. Spectra were acquired on a Bruker Ultraflex –III MALDI‐TOF/TOF mass spectrometer (Bruker Daltonics, Bremen, Germany) with acquisition in MS reflection mode. In all cases, 1600 spectra were summed over the m/z range 500–5000. The supernatant and the organic‐extracted fraction from supernatant were evaluated by MALDI‐ToF MS. All analyses were carried out in triplicate in comparison with a controls.

#### Electrospray Ionisation Mass Spectrometry (ESI‐MS)

ESI‐MS was carried out via direct infusion of 1 uL of the sample (supernatant or organic fraction of supernatant) in MicrOTOF−Q III (Bruker Daltonics, Bremen, Germany) at a flow rate of 0.2 mL/min using 50 : 50 MeOH:water at a full scan (m/z 50–1400) in negative and positive ion modes (m/z 50–1400). All analyses were carried out in triplicate in comparison with a controls.

#### High‐Performance Anion‐Exchange Chromatography Coupled with Pulsed Amperometric Detection (HPAEC‐PAD)

The release of cello‐oligosaccharides in reactions with AfLPMO9A, PASC, and PET was evaluated by HPAEC‐PAD23. Peaks of native cello‐oligosaccharides (Glc2–Glc6) were assigned based on standards from Megazyme Inc. All experiments were carried out in triplicate in comparison with a controls.

### Synergy Assays

The synergism between *Af*LPMO9A and *Is*PETase was evaluated by the sequential or simultaneous addition of enzymes to reactions containing PET powder as substrate (8 mg). PET powder was obtained by milling the commercial PET bottle in a ball mill machine (Tecnal, São Paulo, Brazil). In sequential assays, PET was pretreated with *Af*LPMO9A (0.5, 2 or 4 μM) obeying the same conditions highlighted in “Enzyme assays‐LPMOs”. The residual substrates were i, 3x (washed with Milli‐Q water followed by sonication [10–15 min each]) or ii, 3x (washed with SDS 2 % for 1 h 45 min at 50 °C, sonicated for 15 min, rinsed with water, washed with ethanol for 30 min at 30 °C/300 rpm, followed by washing with water) to remove the bound protein fraction. The residual substrates from control reactions (“no LPMO treatment”) were washed depending on whether washing procedure “i” or “ii” were applied to the treatments. Then, the washed residual substrates were placed in a clean Eppendorf tube where *Is*PETase (0.05 μM) was added and the reactions were carried out in 0.05 M sodium phosphate buffer, pH 7.2, at 30 °C/400 rpm/120 h. The same protocol was used in reactions with BSA and LD. In the simultaneous assays, *Af*LPMO9A and *Is*PETase were added simultaneously to reactions containing PET. 4 μM *Af*LPMO9A were added per reaction. The *Is*PETase concentration was maintained fixed at 0.05 μM in 0.05 M sodium phosphate buffer, pH 6.0, 37 °C/850 rpm/120 h. All the controls used for the sequential assays were adopted for the simultaneous assays.

The supernatants from sequential and simultaneous assays were heated to 95 °C for 5 min and dried in SpeedVac concentrators (Eppendorf, Enfield, CT, USA). The remaining powder was resuspended in a mixture of 20 % DMSO in methanol, solubilized in an ultrasound bath, homogenised in vortex and filtered using 0.22 μm syringe filters (Millex). The concentration of BHET, MHET and TPA released by *Is*PETase was determined by high performance liquid chromatography (HPLC). Reactions with the addition of only *Is*PETase were adopted as controls for both simultaneous and sequential assays. All reactions were carried out in a volume of 600 μl and triplicate.

The boosting effect on PETase activity promoted by the pre‐treatment of PET with BSA or LD was evaluated following the same protocol adopted for *Af*LPMO9A.

### BHET, MHET and TPA Quantification

BHET, MHET and TPA were quantified in liquid chromatograph Ultimate 3000 (Thermo Fisher Scientific, Waltham, MA, USA) equipped with an UV detector at 240 nm using an Acclaim C18 column (Thermo Fisher Scientific, Waltham, MA, USA) at 40 °C using the method described in^[26]^ with modifications. The samples were run following a gradient of 20 mM phosphoric acid (A) and methanol (B): 0–5 min: 20 % B and 80 % A; 5–20 min: 20–65 % B and 80–35 % A; 20–20.01 min: 65–20 % B and 35–80 % A; 20.01–25 min: 20 % B and 80 % A at a flow of 0.6 mL/min. The products of *Is*PETase activity were quantified using a standard curve with known concentrations of BHET, MHET and TPA.

### MHET Synthesis

BHET and TPA are sold by Sigma‐Aldrich (St. Louis, MO, USA). MHET was produced by the partial hydrolysis of BHET with KOH^[26]^: BHET, 11.06 g; KOH, 2.36 g; and distilled ethylene glycol, 90 mL, were mixed and put in glycerine bath to maintain the temperature between 116–118 °C at 80 rpm during 2 h 30 min. Then, the mixture was cooled and transferred to a decanting funnel and extracted with 5 mL dichloromethane three times. The pH of the aqueous phase was adjusted to pH 3.0 with 25 % HCl, cooled at −6 °C for 1 h 30 min and filtered in a glass filter (G3). The liquid fraction was discarded and 30 mL of boiling water was added to the precipitate followed by filtration. The residual precipitate was boiled and filtered. The liquid fraction was cooled at −6 °C for MHET precipitation and filtered in a glass filter. The solid fraction containing the MHET was washed to remove any impurities and dried at 60 °C. A total of 3.1 g MHET (yield of 28 %) at 93.5 % purity was obtained.

The chemical composition of the MHET obtained was confirmed by nuclear magnetic resonance (NMR). ^1^H NMR spectra of samples in DMSO‐d6 were acquired using an Agilent DD2 NMR Spectrometer (Agilent Technologies Inc., Santa Clara, CA, USA) equipped with a triple resonance probe operating at a ^1^H resonance frequency of 499.726 MHz and constant temperature of 298 K (25 °C). A total of 16 free induction decays were collected with 32‐k data points over a spectral width of 16 ppm using a relaxation delay of 1.5‐s. Tetramethylsilane was used as reference for calibration at 0.0 ppm chemical shift.

### Detection of Protein Bound to PET

In addition to XPS, the amount of protein bound to PET was indirectly estimated by measuring the protein that remained in the soluble fraction (supernatant) after incubation with 4 μM *Af*LPMO9A, BSA, or LD in 0.05 M sodium phosphate buffer, pH 6.0, at 37 °C/850 rpm/48 h. After 48 h, the samples were centrifuged at 4 °C/12,000 rpm/30 min, and the supernatant was taken and read at 280 nm in spectrophotometer. Control reactions adopted in this experiment include: i, reactions with substrate in the absence of enzyme (blank); ii, reactions with enzyme but without substrate.

### Statistical Analysis

Data were analysed by ANOVA and Tukey′s test at 5 % significance level.

### Hydrophobicity Analysis

The hydrophobicity average of HFBII from *Trichoderma reesei* (PDB code: 2B97) (a)*, Af*LPMO9A (7OVA) (b), *Kp*LPMO10 A (6NDQ), BSA (4F55), and LD (AlphaFold structure downloaded from Uniprot database [P19858]) were calculated using the Kyte‐Doolittle scale in Chimera^[27]^ and as the GRAVY index score using the ExPASy‐ProtParam Tool (https://web.expasy.org/protparam/).

## Results and Discussion

### AA9 LPMO Pre‐Treatment of PET Increases Subsequent Action by a PETase, but not by Oxidative Modification of the PET Polymer

We investigated the ability of a LPMO to boost the action of a known PETase from the bacterium *I. sakaiensis* ^[9]^ on PET in two separate assays. In the first, a milled PET bottle sample (Figure S1a) was pre‐treated with a solution of *Af*LPMO9A before removal of any adsorbed LPMO by washing with H_2_O under sonication. In the second, given the pH and temperature compatibility of *Is*PETase and *Af*LPMO9A,^[28]^ a combined solution of *Af*LPMO9A and *Is*PETase was added to PET samples. For both experiments, an *Af*LPMO9A from the fungus *Aspergillus fischeri* was used, which has activity on cellulose (phosphoric acid swollen cellulose ‐ PASC, C1/C4) and hemicellulose (xyloglucan ‐ XG) (Figure S2).

**Figure 1:**
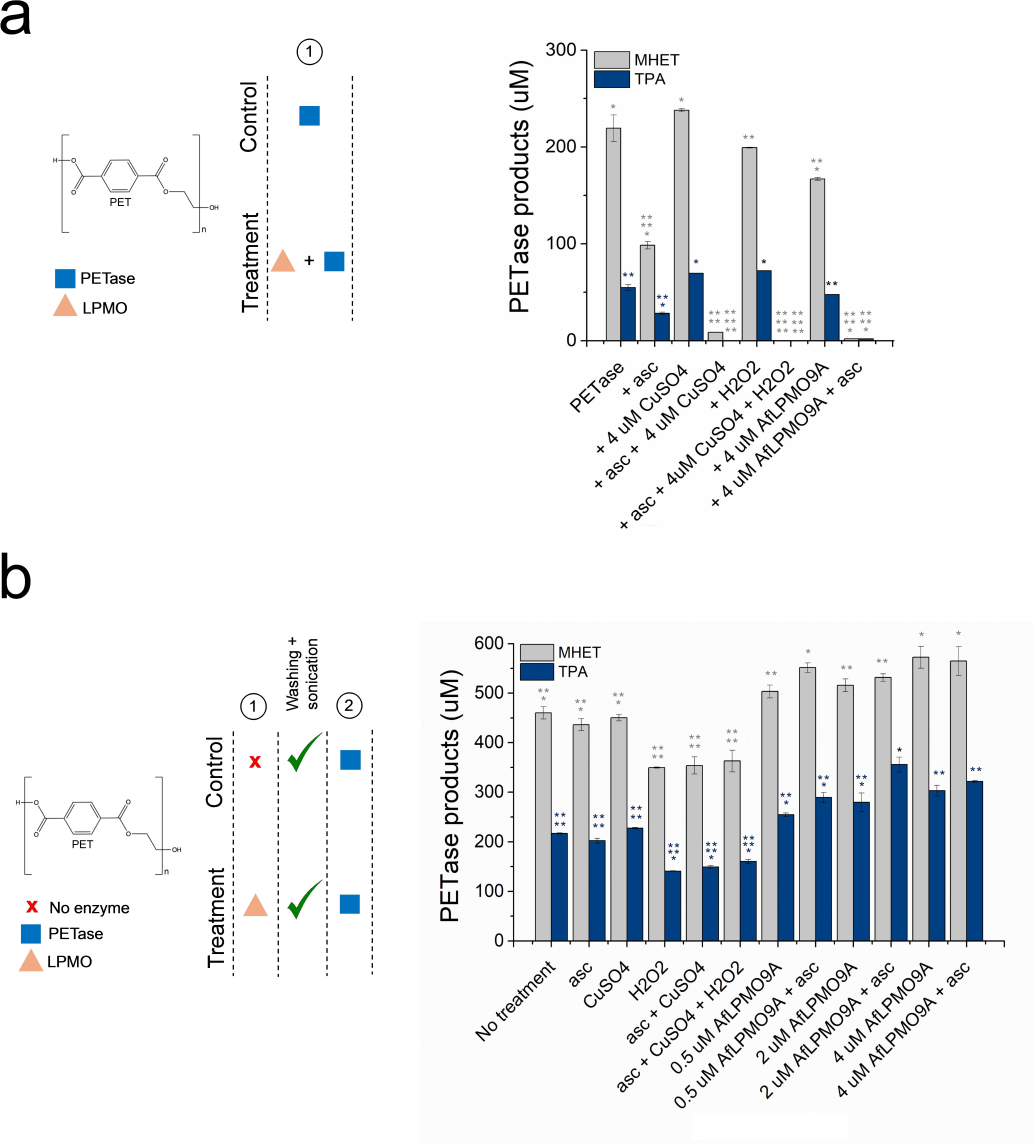
MHET and TPA detected in reactions with the simultaneous (a) or sequential addition (b) of *Af*LPMO9A and PETase to PET. Control reactions include ascorbate (1 mM), CuSO_4_ (4 μM), H_2_O_2_ (100 μM), ascorbate+CuSO_4_, and ascorbate+CuSO_4_+H_2_O_2_. Reactions with the simultaneous addition of *Af*LPMO9A and *Is*PETase were incubated in 0.05 M sodium phosphate buffer, pH 6.0, at 37 °C for 120 h. The pre‐treatment of milled PET bottle with *Af*LPMO9A were carried out in 0.05 M sodium phosphate buffer, pH 6.0, at 37 °C for 48 h/850 rpm, followed by three steps of sonication in MilliQ water (10–15 min) of the residual substrate and addition of *Is*PETase (0.05 M sodium phosphate buffer, pH 7.2, at 30 °C for 120 h/400 rpm). The same concentration of *Is*PETase was used in both assays (0.05 μM). Bars followed by the same symbol do not differ according to Tukey‘s test at 95 % confidence level.

For the latter assay, it was found that addition of a combined solution of *Af*LPMO9A and *Is*PETase to PET actually lowered the esterase activity when compared to the addition of *Is*PETase alone to PET, particularly in the presence of ascorbate (Figure 1a and Figure S3). We presume that this observation shows that LPMOs and/or ascorbate can degrade the PETase, through undefined redox chemistry on the protein, highlighting the known deleterious effects of reactive oxygen species which are generated by LPMOs in the absence of a natural substrate.^[29]^ In accord with this proposal, whereas the simultaneous addition of copper (CuSO_4_) and *Is*PETase to PET resulted in a slight increase in the production of the MHET and TPA, a combination of ascorbate+CuSO4, and ascorbate+CuSO_4_+hydrogen peroxide (H_2_O_2_) lowered the MHET and TPA production by 70 % and 100 %, respectively. As such, no convincing evidence was found that the simultaneous addition of *Af*LPMO9A and *Is*PETase to PET boosted the action of *Is*PETase.

In contrast, for the former assay the pre‐treatment of milled PET bottle with *Af*LPMO9A and then washing and sonication of the residual substrate to remove any LPMO, followed by addition of *Is*PETase to the PET substrate improved the relative release by *Is*PETase of MHET and TPA by up to 24–64 %, respectively (Figure 1b). Mindful that the apparent boosting could arise from Fenton‐like chemistry from the combination of ascorbate, oxygenated buffer and adventitious metal ions, several control experiments were performed as follows: reaction of the substrate with ascorbate, CuSO_4_, H_2_O_2_, ascorbate+CuSO_4_, and ascorbate+CuSO_4_+H_2_O_2_; none of which resulted in any boosting on MHET and TPA release by *Is*PETase (Figure 1b).

Given the ostensible synergy between *Af*LPMO9A and *Is*PETase when the two enzymes are added sequentially (Figure 1b), we sought to determine the mechanism of action and to investigate whether the increased PETase activity was due to the prior oxidative modification of the PET by the LPMO. Oxidation of the polymer was therefore evaluated through matrix‐assisted laser desorption/ionisation time‐of‐flight mass spectrometry (MALDI‐ToF MS) of the PET, before and after LPMO action. However, no products as a result of LPMO action were detected using this method. Moreover, incubation of the *Af*LPMO9A reaction supernatant with *Is*PETase did not result in any detectable amounts of BHET, MHET, TPA by high performance liquid chromatography (HPLC). A soluble product was detected by electrospray ionisation mass spectrometry (ESI‐MS *m/z* 165.0406, Figure S4) in the supernatants of a mixture of *Af*LPMO9A and milled PET. An extensive search of molecules with this molecular mass^[30]^ gave D‐xylonate (C_5_H_9_O_6_, M^−1^=165.0406) as the only plausible product (Figure S5). In explaining the unexpected appearance of this species, we note that D‐xylonate is an intermediate used in the biosynthesis of ethylene glycol from renewable sources, in a technology adopted by Coca‐Cola company since 2009.[^31^, ^32^] Its appearance therefore is likely to be a remnant of bio‐based ethylene glycol production, and that the LPMO releases the D‐xylonate from this form of PET, possibly by the production of hydrogen peroxide given that the same peak is also found in control reactions containing ascorbate, CuSO_4,_ and ascorbate+CuSO_4_. This is supported by the fact that the peak at the corresponding *m/z* is no longer observed in reactions with milled PET Mylar as the substrate (data not shown).

**Figure 2:**
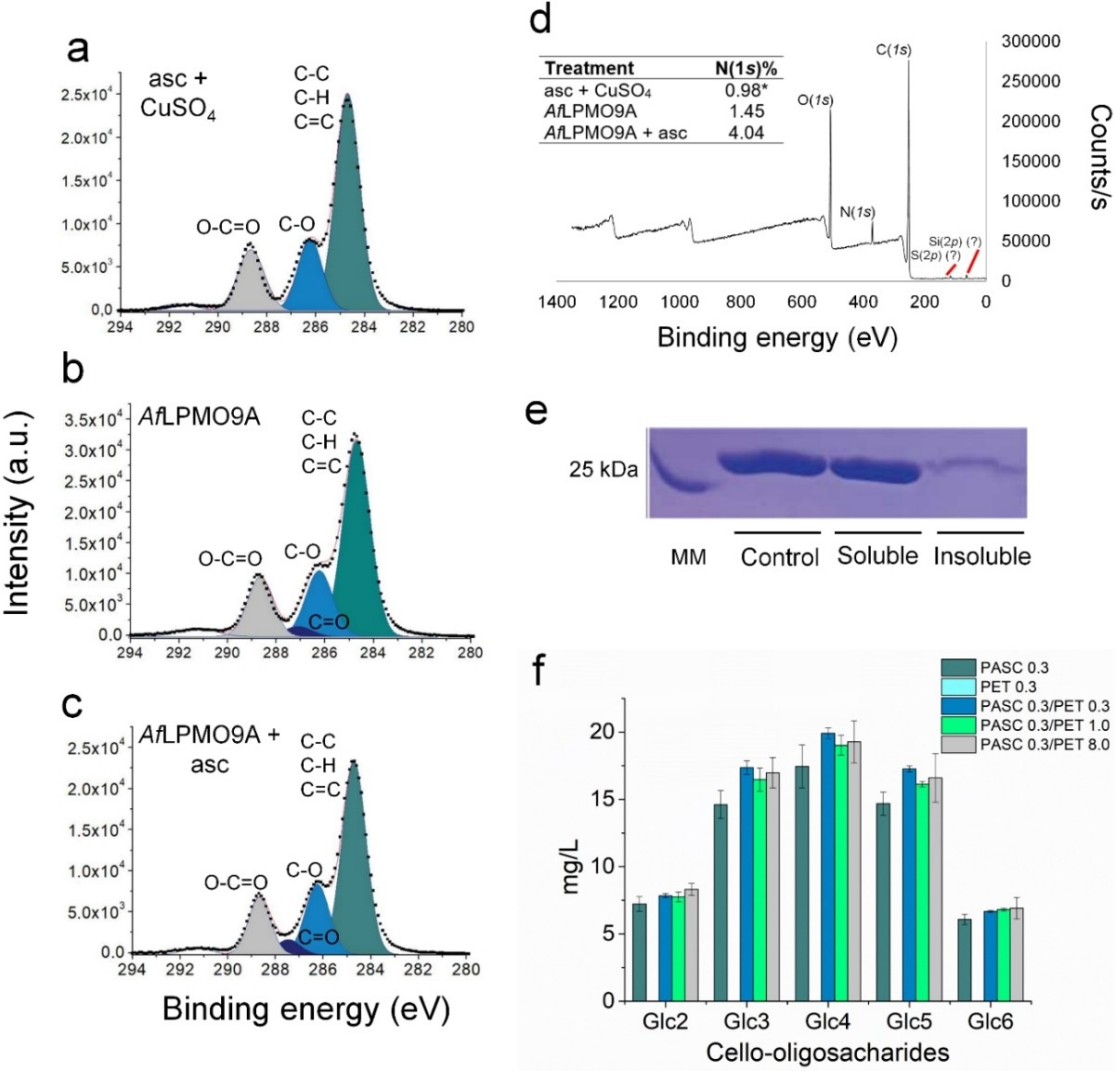
C(1 *s*) XPS spectra of ascorbate+CuSO_4_ ‐ control (a), *Af*LPMO9A (b), and *Af*LPMO9A+ascorbate‐treated PET bottle coupons (c): 284.7 eV, C−C/C=C/C−H; 286.2 eV, C−O; 287.5 eV, C=O, 288.6 eV, O−C=O. XPS survey spectra of *Af*LPMO9A+ascorbate‐treated PET bottle coupons (d). The peak corresponding to N atoms is verified at 400 eV. The table shows the nitrogen detected in the control and *Af*LPMO‐treated samples. Binding of *Af*LPMO9A to PET bottle coupon evaluated by SDS‐PAGE (e). Cellulosic substrate (PASC) and PET do not compete for the binding of *Af*LPMO9A (f). The ascorbate+CuSO_4_, *Af*LPMO9A, *Af*LPMO9A+ascorbate reactions with PET coupons were carried out in 0.05 M sodium phosphate buffer, pH 6.0, at 37 °C/850 rpm for 48 h. For the binding assays, *Af*LPMO9A was incubated (0.05 M sodium phosphate buffer, pH 6.0) with PET bottle coupon on ice and the binding to the substrate was evaluated as recommended in.^[36]^ Control, incubation of *Af*LPMO9A without substrate; soluble, *Af*LPMO9A in the supernatant of reactions with PET; insoluble, *Af*LPMO9A bound to milled PET. The *Af*LPMO9A+ascorbate reactions with PASC (0.3 mg/mL) and different concentrations of PET (0.3, 1.0, 8.0 mg/mL) were carried out in 0.05 M sodium phosphate buffer, pH 6.0, at 37 °C/850 rpm for 16 h. The asterisks mean that the quantification is below the equipment limits detection. asc, ascorbate.

Given the lack of any detectable small PET‐based ‘oligomeric’ products by MS and HPLC, we turned to X‐ray photoelectron spectroscopy (XPS) to investigate if the action of *Af*LPMO9A had chemically modified the PET surface,^[33]^ but without the concomitant release of oxidised oligomeric fragments. The C(1 *s*) (Figure 2a–c and Table 1) and O(1 *s*) XPS spectra (Figure S6) of PET bottle coupons treated with *Af*LPMO9A or *Af*LPMO9A+ascorbate, and then washed under sonication, did indeed exhibit an increased presence of C=O groups (287.5 and 531.5 eV respectively) compared to the control. Whilst this observation is in accordance with an oxidative modification of PET surface by LPMO, there is also the possibility that some *Af*LPMO9A remained bound to the PET despite the thorough washing procedure, leading to the appearance of C=O groups in the XPS data. As such, the increase in XPS signals due to C=O groups could arise from the carbonyl groups of any surface‐bound protein, which would be expected to have signals in similar regions (we note here that the O(1 *s*) signal increase at 531.5 eV falls within the range expected for amide carbonyls, rather than aliphatic carbonyl groups, suggestive of the signal arising from the amide carbonyls of the adsorbed protein).^[34]^ In accord with this possibility, the intensity of the peak for C=O was unaffected by the presence/absence of ascorbate in the reaction mixture, and – more tellingly – there was a commensurate increase in the intensity of a peak at 400 eV in the low resolution XPS spectrum, associated with the presence of N atoms in the sample (Figure 2d). Thus, the XPS data are commensurate with adsorption of LPMO onto the PET surface (Figure 2e and Figure S7a), rather than any oxidative modification of the surface by the enzyme. The XPS data were acquired using PET coupons. To test our hypothesis that the binding of LPMO to PET observed with XPS can be extended to the conditions used in the synergy assays, we incubated milled PET with *Af*LPMO9A and analysed by the amount of enzyme that remained in the soluble fraction (supernatant) after incubation. Nearly 100 % of the LPMO was detected in reactions without the reductant, while the addition of ascorbate increased the binding of LPMO to PET by 50 % (Figure S8), corroborating the XPS data and the boosting effect observed in the synergy assays.

**Table 1:** Abundance (%) of C(1 *s*) components on PET surface.

	Treatments		
Binding energy (eV)	asc+CuSO_4_	*Af*LPMO9A	*Af*LPMO9A+asc
288.6 (O−C=O)	18.23±1.23	16.41±1.34	15.46±0.76
287.5 (C=O)	x	3.02±0.66	4.44±0.23
286.2 (C−O)	19.64±0.90	19.40±0.89	20.82±1.23
284.7 (C−C/C−H/C=C)	59.56±2.67	57.83±1.98	56.97±0.12
291.7*	2.57±0.03	3.34±0.24	2.31±0.43

*π‐π* shake‐up transition associated with the aromatic ring; asc, ascorbate.

In the knowledge that the LPMO adsorbs to the surface of the polymer, we tested the effects of adding milled PET (0.3–8.0 mg/mL) on the reaction of *Af*LPMO9A with the known substrate, PASC, to see if the milled PET could act as a competitive inhibitor of the reaction of active LPMOs with a polysaccharide (Figure 2f). Here, no significant difference was seen in the production of cello‐oligosaccharides in the presence of milled PET, showing that any interaction of milled PET with catalytically active *Af*LPMO9A is weak or non‐existent. This observation points us towards the likelihood that any protein adsorbed onto the PET surface, as seen by XPS, is likely to be denatured protein *already* present in the sample. Put another way, the presence of PET affects the natural equilibrium which exists between folded and unfolded states of the protein, where the latter is removed from the solution by deposition onto the polymer surface, eventually leading to full denaturing of the protein.

Thus, we conclude that the increased presence of peaks assignable to the C=O group arise from the irreversible deposition of likely‐denatured LPMO protein onto the polymer surface, which could not be removed even after aqueous washing and sonication steps.^[35]^

### AA9 LPMO Boosts PETase Activity through a Non‐Catalytic Pathway

The appearance of LPMO protein, albeit denatured, on the surface of PET led us to re‐hypothesise that *Af*LPMO9A boosts PETase activity through a non‐catalytic pathway, akin to that seen for the action of hydrophobins in enhancing the degradation of PET by PETases.[^22^, ^37^] Hydrophobins are small (5–20 kDa) globular proteins produced by filamentous fungi, that self‐assemble into amphipathic layers onto the surface of polymers. As a result, any hydrophobic surface becomes more hydrophilic due to the adsorption of hydrophobins, and through this action it is proposed that hydrolases are drawn from the aqueous phase towards the surface through electrostatic interactions, thus stimulating the hydrolytic breakdown of the substrate.[^37^, ^38^, ^39^, ^40^] In accord with this hypothesis, a previous study of a polysaccharide‐inactive LPMO (AA14 ‐ *Pc*AA14 A) showed enhanced activity for PET degradation when acting simultaneously (as opposed to sequentially) with an *Is*PETase.^[21]^ Moreover, Ribitsch et al.^[41]^ showed that the interaction of a cutinase from *Thermobifida cellulosilytica* with a surface‐bound *Trichoderma sp*. hydrophobin led to enhancement of PET hydrolysis of ~16‐fold. Additionally, in parallel to the results presented herein, Takahashi et al.^[37]^ and Puspitasari et al.^[22]^ demonstrated that the hydrophobin boosting of PETase and cutinase action on PET only occurred when PET was pre‐treated with hydrophobins.

**Figure 3:**
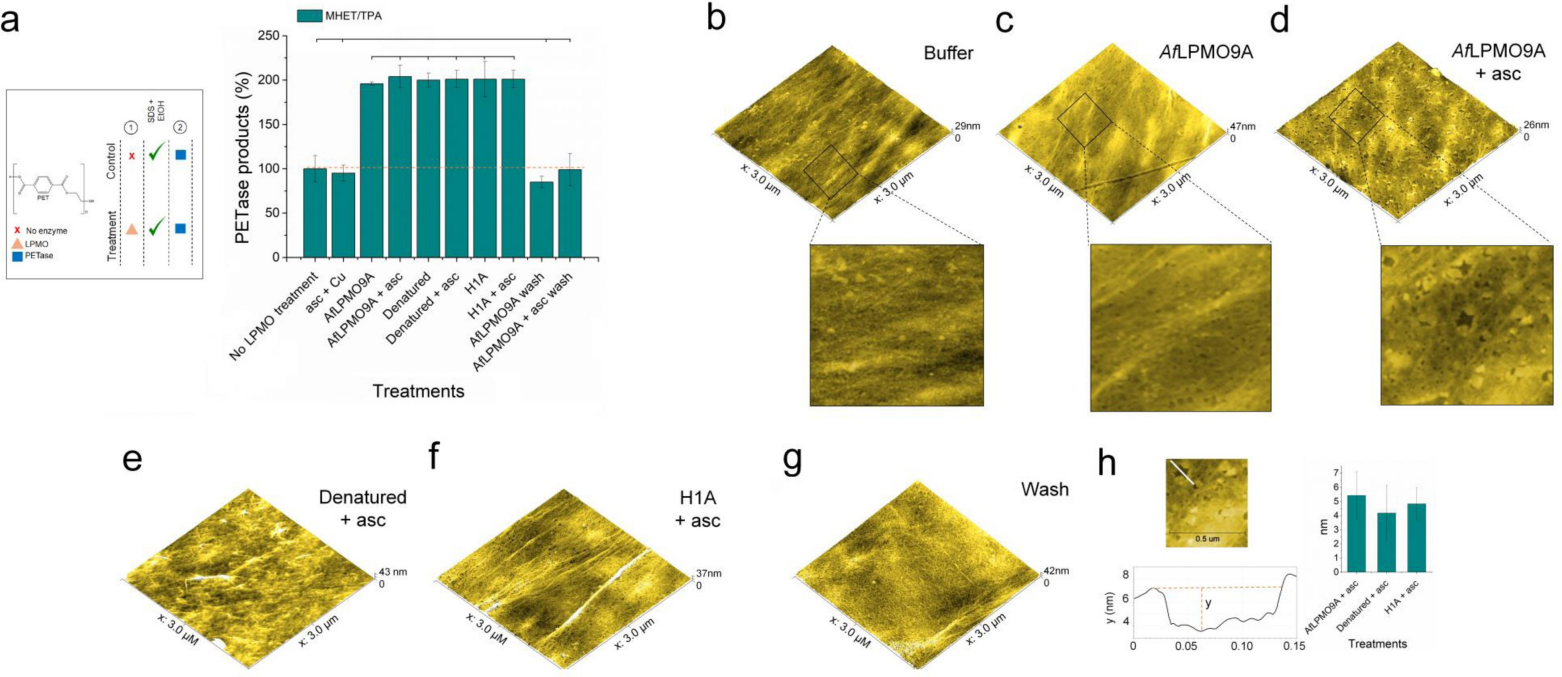
Sequential synergy assays with *Af*LPMO9A, denatured *Af*LPMO9A, and *Af*LPMO9A^H1A^ followed by the addition of *Is*PETase^S121E/D186H/R280A^ (a). “Wash” samples were treated with 3 steps of SDS+sonication+ethanol (See experimental section) after the pre‐treatment with *Af*LPMO9A, denatured *Af*LPMO9A, or *Af*LPMO9A^H1A^. Topographies (3×3 μm) of PET bottle coupons treated with buffer (b), *Af*LPMO9A (c), *Af*LPMO9A+ascorbate (d), denatured *Af*LPMO9A+ascorbate (e), *Af*LPMO9A^H1A^+ascorbate (f) followed by SDS+sonication+ethanol (g). Depth of the spots/depressions found in the surface of *Af*LPMO9A+ascorbate, denatured *Af*LPMO9A+ascorbate, and *Af*LPMO9A^H1A^+ascorbate‐treated PET samples (h). PET bottle coupons were incubated with 4 μM LPMO in 0.05 M sodium phosphate buffer, pH 6.0, for 48 h at 37 °C/850 rpm followed by sonication with MilliQ water or SDS+sonication+ethanol before visualisation by AFM. A more detailed topography of the reactions of PET bottle treated with buffer, *Af*LPMO9A, and *Af*LPMO9A+asc is available in b, c, and d, respectively. Bars followed by the same symbol do not differ according to Tukey‘s test at 95 % confidence level. asc, ascorbate.

In testing this new hypothesis, we used *Af*LPMO9A, its denatured version, and the mutant *Af*LPMO9A^H1A^, in which the key active site histidine residue had been mutated for an alanine, for sequential assays with *Is*PETase^S121E/D186H/R280A^.^[23]^ For the control experiment, we further changed the washing step from simple sonication of the sample to washing with SDS and ethanol, a procedure known to be more aggressive for the removal of surface‐absorbed species. Under these conditions, the same release of MHET and TPA was observed for the wild‐type LPMO, its denatured form and its *Af*LPMO9A^H1A^ mutant (Figure 3a), showing that any boosting on PETase activity promoted by the LPMO is due to the presence of the protein itself, rather than any inherent oxygenase activity. In addition, the inclusion of SDS and ethanol in the washing steps (SDS+sonication+ethanol) in the PET pre‐incubated with *Af*LPMO9A and *Af*LPMO9A+ascorbate reduced the percentage of nitrogen on the PET surface (Table 2) and, at the same time lowered, the levels of MHET and TPA produced by PETase to the ones observed in the control reactions (Figure 3a), showing that sonication in the previous round of experiments (Figure 1b) had not removed all adsorbed LPMO.

**Table 2:** Atomic percentage (at %) of elements obtained by XPS for C, O and N on PET coupon.

	Treatments 3×sonication			
	Buffer	CuSO4+asc	*Af*LPMO9A	*Af*LPMO9A+asc
C(1 *s*)	73.80±0.66	74.12±0.61	72.87±1.88	72.23±2.02
O(1 *s*)	22.49±0.75	22.42±0.5	22.30±1.62	20.62±0.55
N(1 *s*)	1.32±0.31*	1.37±0.17*	**1.74±0.64**	**4.01±0.44**
Others**	2.38±0.023	2.08±0.26	3.09±0.89	3.14±1.91

	3×SDS+sonication+ethanol			
C(1 *s*)	74.37±0.61	73.91±0.25	72.98±0.53	71.90±1.74
O(1 *s*)	25.24±0.31	25.43±0.16	25.77±0.31	24.28±0.32
N(1 *s*)	0.0±0.0*	0.52±0.03*	0.96±0.14*	1.02±0.13*
Others**	0.38±0.033	0.14±0.25	0.29+0	1.07±1.55

*Quantification above the equipment limits; **Si(2*p*), S(2*p*); asc, ascorbate.

We further used atomic force microscopy (AFM) to analyse PET bottle coupons which had been treated, again, with *Af*LPMO9A, denatured *Af*LPMO9A and *Af*LPMO9A^H1A^ followed by sonication or by washing with SDS+sonication+ethanol. The topographies of PET coupons incubated with buffer or ascorbate+CuSO_4_ (controls) showed the inherent roughness of the unmodified polymer and a distribution of fibres on its surface typical for a PET sample (Figure 3b). In contrast, PET treated with *Af*LPMO9A (Figure 3c) and *Af*LPMO9A+asc (Figure 3d), denatured *Af*LPMO9A (Figure 3e) or *Af*LPMO9A^H1A^ (Figure 3f) exhibited a surface topography commensurate with the deposition of a continuous layer of proteins with spots/depressions (4–5 nm depth) randomly distributed over the entire surface (Figure 3g), in what resembles the deposition of hydrophobins on flat surfaces like glass.^[42]^ The same behaviour is seen when PET film (Mylar^®^) was used as substrate in reactions with *Af*LPMO9A (Figure S9). The same pattern is no longer seen if the *Af*LPMO9A‐treated samples were then subsequently washed with SDS+sonication+ethanol (Figure 3h). The AFM data are therefore in accord with LPMO adsorption on the PET polymer surface, as previously suggested from the XPS data described above.

### The Surface Binding Ability of AA9 LPMO is Extended to other Plastics, LPMO Families, and other Enzymes

The finding that LPMOs can bind to hydrophobic polymer surfaces and potentiate any polymer‐active enzymes to polymer degradation, opens‐up the question as to whether LPMOs can bind to polymers other than PET. In this regard, we also investigated whether *Af*LPMO9A could bind to high‐density polyethylene (HDPE) and polypropylene (PP) (Figure S1c–d), since these are polymers of significant environmental concern. HDPE is formed by the polymerization of ethylene units [(C_2_H_4_)_*n*_] while the structural formula of PP is similar to that of polyethylene, except for the substitution of a hydrogen for a methyl as a pendant group.^[43]^

**Figure 4:**
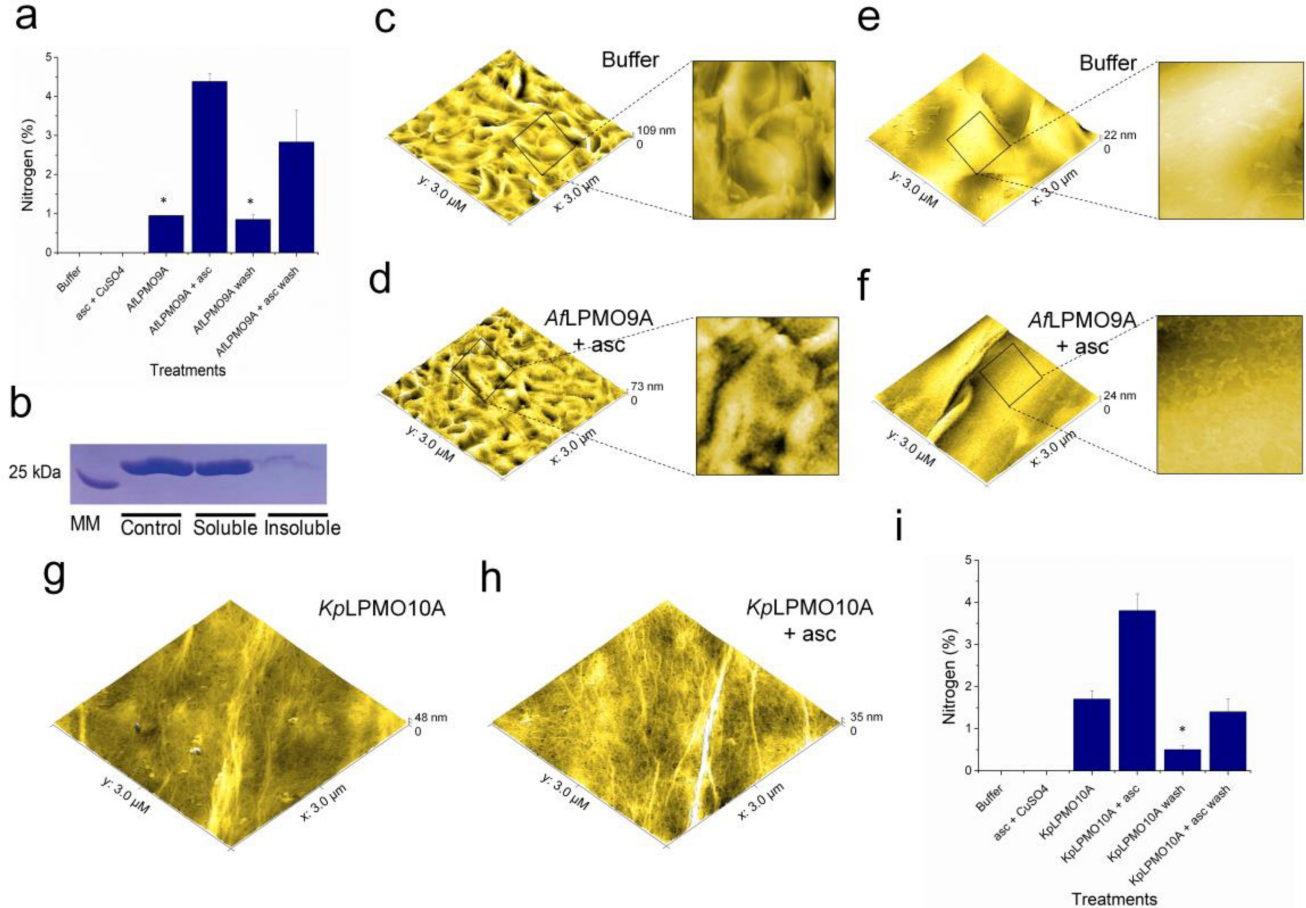
Nitrogen (at %) detected on the surface of HDPE coupons treated with buffer, ascorbate+CuSO_4_, *Af*LPMO9A, *Af*LPMO9A+ascorbate (sonication or SDS+sonication+ethanol treatments) (a). Binding of *Af*LPMO9A to milled HDPE evaluated by SDS‐PAGE (b). Topographies (3×3 μm) of HDPE coupons treated with buffer (c), and *Af*LPMO9A+ascorbate (d). Topographies (3×3 μm) of PP coupons treated with buffer (e), and *Af*LPMO9A+ascorbate (f). Topographies (3×3 μm) of PET bottle coupons treated with *Kp*LPMO10 A (g), and *Kp*LPMO10 A+ascorbate (h). HDPE, PP and PET bottle coupons were incubated with 4 μM LPMO in 0.05 M sodium phosphate buffer, pH 6.0, for 48 h at 37 °C/850 rpm followed by sonication before visualisation by AFM. The asterisks mean that the values are below the equipment limits. For the binding assays, *Af*LPMO9A was incubated (0.05 M sodium phosphate buffer, pH 6.0) with milled HDPE on ice and the binding to the substrate was evaluated as recommended in.^[33]^ Control, incubation of *Af*LPMO9A without substrate; soluble, *Af*LPMO9A in the supernatant of reactions with PET; insoluble fraction, *Af*LPMO9A bound to milled PET. A more detailed topography of the reactions of HDPE and PP treated with buffer, and *Af*LPMO9A+asc is available in c, d, e and f, respectively. asc, ascorbate.

XPS data indicate that the incubation of HDPE coupons with *Af*LPMO9A leads to the appearance of N atoms on the surface of the polymer in comparison with the controls (Figure 4a). *Af*LPMO9A remains bound to the insoluble fraction of the reaction with milled HDPE (Figure 4b and Figure S7b) and is found deposited on the entire surface of the polymer in circular shapes (Figure 4c–d). As observed for PET, the inclusion of additional steps of washing lowered the nitrogen concentration in *Af*LPMO9A+ascorbate‐treated samples from 4.3–2.2 % (Figure 4a). The same pattern of protein aggregation was found when PP was treated with *Af*LPMO9A+ascorbate revealing that LPMO does indeed bind to different types of hydrophobic polymers (Figure 4e–f).

In addition to the AA9 LPMO, we investigated whether other LPMO families were also able to bind synthetic polymers like PET. AFM topographies of PET coupons incubated with an AA10 isolated from the bacterium *Kitasatospora papulosa* (*Kp*LPMO10 A) that acts on the polysaccharides cellulose (C1/C4), chitin and (C1) xylan (C4)^[23]^ confirm that *Kp*LPMO10 A binds to PET, following a similar pattern of a protein film distributed on the surface of the polymer (Figure 4g–h). XPS further confirmed the deposition of AA10 on the substrate and that the %N lowers after washings with SDS+sonication+ethanol like observed for the AA9 LPMO (Figure 4i). Taken together, these data show that the binding of LPMOs to synthetic substrates is a general phenomenon.

**Figure 5:**
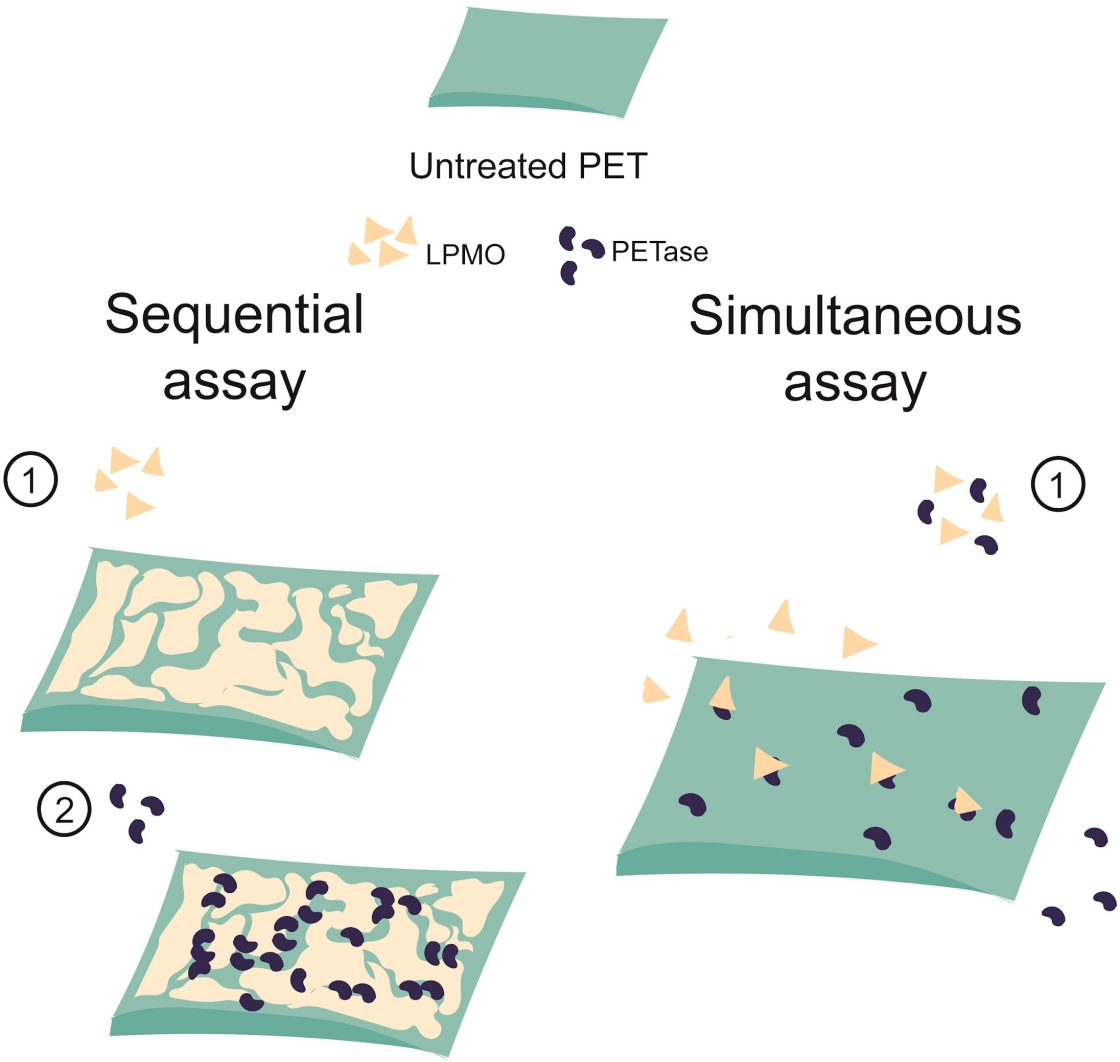
LPMO boosts PETase activity through a non‐catalytic pathway akin to that observed for hydrophobins. In sequential assays (left), the pre‐treatment of PET coupons with LPMO for 48 h favours the binding of *Af*LPMO9A on the surface of the polymer (1) paving the way for PETase activity, resulting in the improvement of MHET and TPA production (2). In simultaneous assays (right), where LPMO and PETase are added simultaneously to the reaction, LPMOs and/or ascorbate can degrade the PETase, through an undefined redox chemistry on the protein, and no synergy is observed.

By analogy, we propose that the deposition of *Af*LPMO9A or *Kp*LPMO1 A exposes its hydrophilic residues paving the way for enhanced PETase activity (Figure 5 and Figure S10). Indeed, molecular dynamics simulations of a biological system co‐displaying hydrophobin and PETase activities showed that the enrichment of PET molecules in the hydrophobic patches of hydrophobin surface favoured PETase activity.^[44]^

Given the hydrophobin effect of LPMOs on PET and other plastics like HDPE and PP, we wondered whether this is an exclusive feature of LPMOs or if it could be extended to other enzyme families. In this context, we are familiar with the positive effects of bovine serum albumin (BSA) on the activity of enzymes targeting the lignocellulose.[^45^, ^46^] Pre‐treating PET with BSA indeed boosts PETase activity in sequential assays (Figure S11). Additionally, washing steps with SDS, sonication and ethanol lowered PETase activity in a manner similar to what was observed for *Af*LPMO9A (Figure S11). Similar to the findings of this paper, a patent from PETROBRAS, a Brazilian company in the oil refinement sector, claims that the pre‐treatment of PET filaments with BSA results in a boosting of PET hydrolase activity.^[47]^ Some other studies, however, have indicated that BSA has esterase activity under specific conditions,[^48^, ^49^] bringing into question its reliability as a control enzyme for PET‐ase activity. As an alternative, the same patent reveals that employing a mixture of cellulases in the pre‐treatment of PET results in an improvement of 33 % in TPA production, suggesting that the hydrophobin effect is not exclusive to LPMOs. In this sense, to broaden the spectrum of enzymes with non‐catalytic effects on synthetic polymers degradation, we also evaluated the role of a protein unrelated to carbohydrate degradation – lactate dehydrogenase (LD) – on esterase activity. As expected, when applied in the pre‐treatment of PET, LD enhanced PETase activity. Additionally, both washing procedures were ineffective at removing the LD bound to PET (Figure S11 and S12), highlighting how the hydrophilic or hydrophobic nature of a protein influences its interaction with synthetic polymers surfaces.

## Conclusions

In the work described herein, we were unable to observe the formation of any oxidised products from PET following the action of *Af*LPMO9A. We re‐hypothesized, based on XPS and AFM data from *Af*LPMO9A and its catalytically inactive mutant (removal of the copper‐binding histidine residues), that the observed boosting of PETase following the pre‐treatment by a LPMO was due to a known hydrophobin effect, in which the LPMO absorbs onto PET surface and recruits PETases towards the polymer surface.

Our understanding of bio‐strategies for plastic upcycling is still in its infancy. While unexpected from a catalytic point‐of‐view, the hydrophobin effect from LPMOs and other enzymes adds to the available biotechnological methods for enhancing the action of PETases on PET[^48^, ^49^] and raises questions on the contribution of the non‐catalytic effects on enzyme systems operating in synergy. Additionally, the use of biomolecules to bind to synthetic polymers like polyethylene and polypropylene could potentially augment the action of other catalysts in the future, and thus offer a further means by which the bioremediation of a wider group of synthetic polymers can be addressed.

## Note Added in Proof

For completeness of the scientific record we note that the following article, dealing with a similar topic to the work presented herein, was published on‐line on September 27, 2024. https://pubs.acs.org/doi/full/10.1021/cbe.4c00125

## Conflict of Interests

The authors declare that a related patent application has been filed under the following application numbers: 21807602.4, US 2023/0193218 A, and BR 10 2021 009404 4.

1

## Supporting information

As a service to our authors and readers, this journal provides supporting information supplied by the authors. Such materials are peer reviewed and may be re‐organized for online delivery, but are not copy‐edited or typeset. Technical support issues arising from supporting information (other than missing files) should be addressed to the authors.

Supporting Information

## Data Availability

The data that support the findings of this study are openly available in Research Data York at https://doi.org/10.15124/71c0a07a‐74d0‐4dee‐aac9‐0ef2f05756af and in the supporting information.
