# Supplementary material for: On the Non‐Catalytic Role of Lytic Polysaccharide Monooxygenases in Boosting the Action of PETases on PET Polymers
Source: ChemSusChem. 2024 Nov 14;18(4):e202401350. doi: 10.1002/cssc.202401350 (PMC11826121; doi:10.1002/cssc.202401350)
Supplement: Supplementary file 1 — Supporting Information [file CSSC-18-e202401350-s001.pdf]

ChemSusChem

Supporting Information

On the Non-Catalytic Role of Lytic Polysaccharide Monooxygenases in Boosting the Action of PETases on PET Polymers

Thamy L. R. Corrêa,* Ellen K. B. Román, Carlos A. R. Costa, Lucia D. Wolf, Richard Landers, Peter Biely, Mario T. Murakami, and Paul H. Walton*

Supporting information

On the non-catalytic role of lytic polysaccharide monooxygenases in boosting the action of PETases on PET polymers

Thamy L. R. Corrêa[1*], Ellen K. B. Román[2], Carlos A. R. Costa[3], Lucia D. Wolf[4], Richard Landers[5], Peter Biely[6], Mario T. Murakami[4], Paul H. Walton[1*]

[1]Department of Chemistry, University of York, Heslington York, YO10 5DD, United Kingdom.

[2]Molecular and Morphofunctional Biology Graduate Program, Institute of Biology, University of Campinas (UNICAMP), Campinas, Brazil.

[3]Brazilian Nanotechnology National Laboratory (LNNano), Brazilian Center for Research in Energy and Materials (CNPEM), Campinas, Brazil.

[4]Brazilian Biorenewables National Laboratory (LNBR), Brazilian Center for Research in Energy and Materials (CNPEM), Campinas, Brazil.

[5]Institute of Physics Gleb Wataghin, University of Campinas (UNICAMP), Campinas, Brazil.

[6]Institute of Chemistry, Slovak Academy of Sciences, Bratislava, Slovak Republic.

*Corresponding authors: paul.walton@york.ac.uk; thamy.correa@york.ac.uk

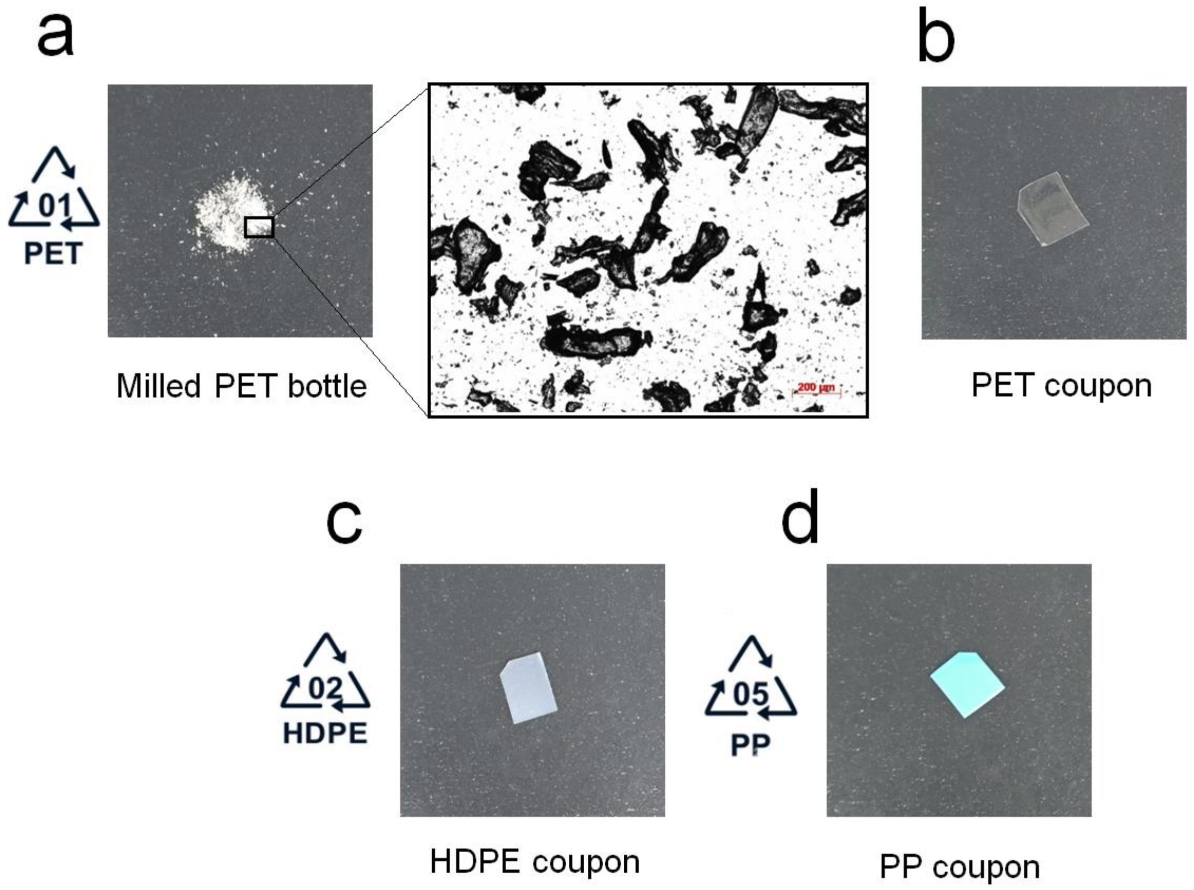

Figure S1. Plastic polymers used in this work. Microscopic view of the fragments of PET obtained after milling PET bottle coupons (a), PET bottle coupon (0.6 x 0.4 cm) (b), HDPE coupon (0.6 x 0.4 cm) (c), and PP coupon (0.6 x 0.4 cm). The milled PET bottle (a) used in this work has dimensions compatible with microplastics.

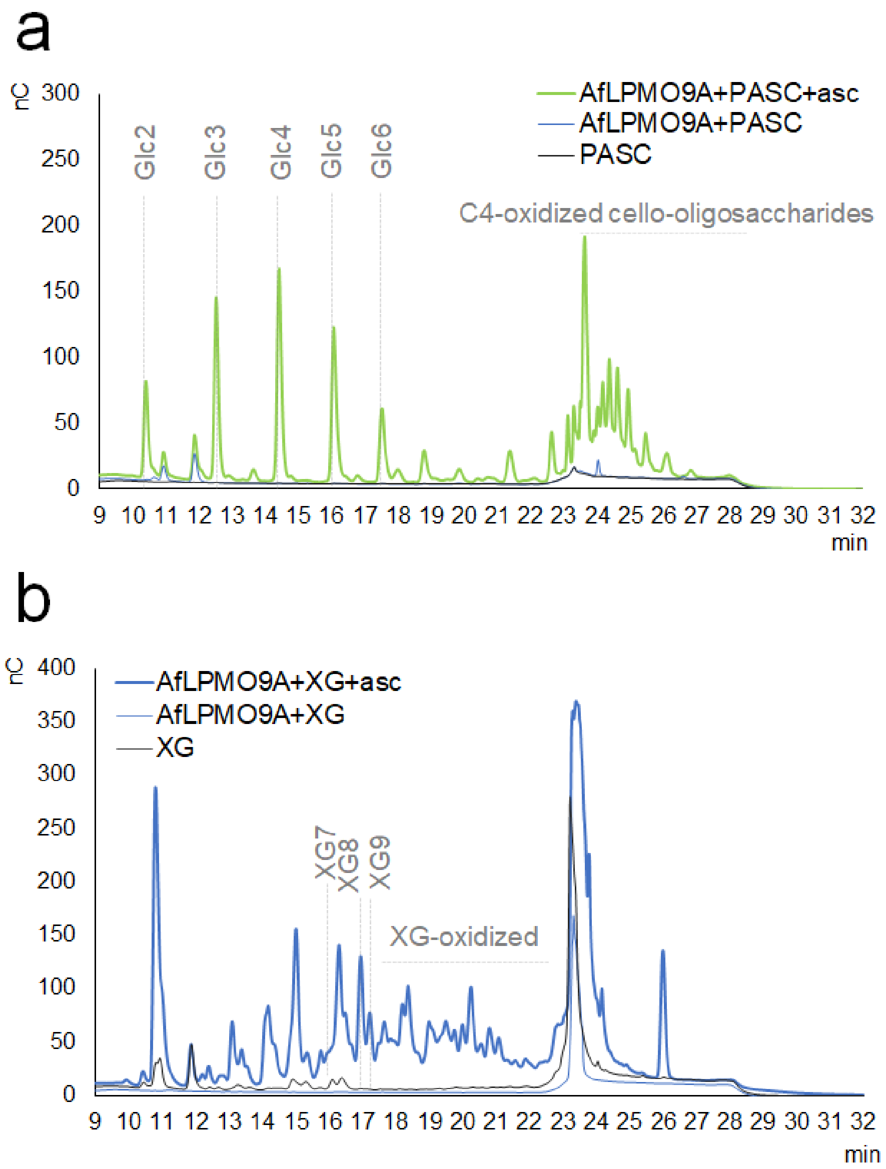

Figure S2. Activity of *Af*LPMO9A on PASC and XG. HPAEC-PAD profile of native and oxidized products released from PASC (a) and XG (b) by *Af*LPMO9A. Reactions were carried out at pH 5.0/50 ºC for 16 h in the presence or absence of the reductant - ascorbate. Control assays include: i, buffer and substrate; ii, buffer, and ascorbate in the absence of *Af*LPMO9A. Native and oxidized sugars were assigned based on standard oligosaccharides and previous works, respectively[1,2]. asc, ascorbate; nC, nanocoulomb.

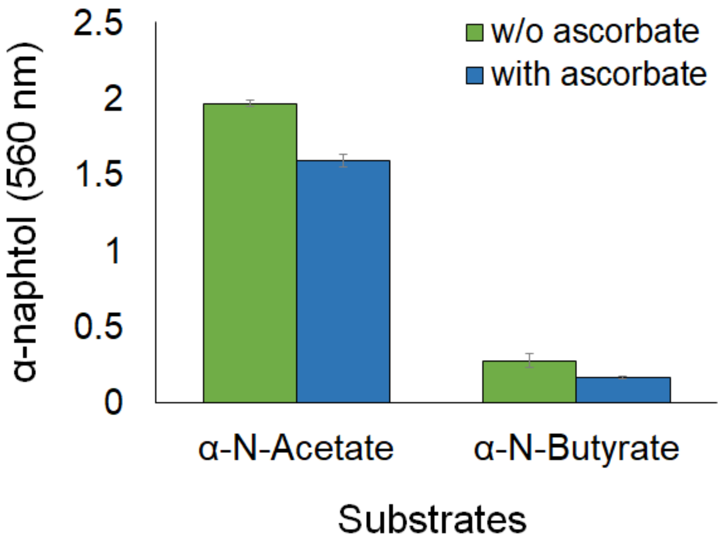

Figure S3. Activity of *Is*PETase on α-naphthyl acetate and α-naphthyl butyrate. The esterase activity of *Is*PETase was determined by incubating 0.05 µM *Is*PETase with 4 mM α-naphthyl acetate or α-naphthyl butyrate at pH 6.0 in 0.05 M sodium phosphate buffer at 37 °C/400 rpm/60 min. The production of α-naphtol (560 nm) was detected following[3]. The effect of ascorbate on *Is*PETase activity was measured at 1mM.

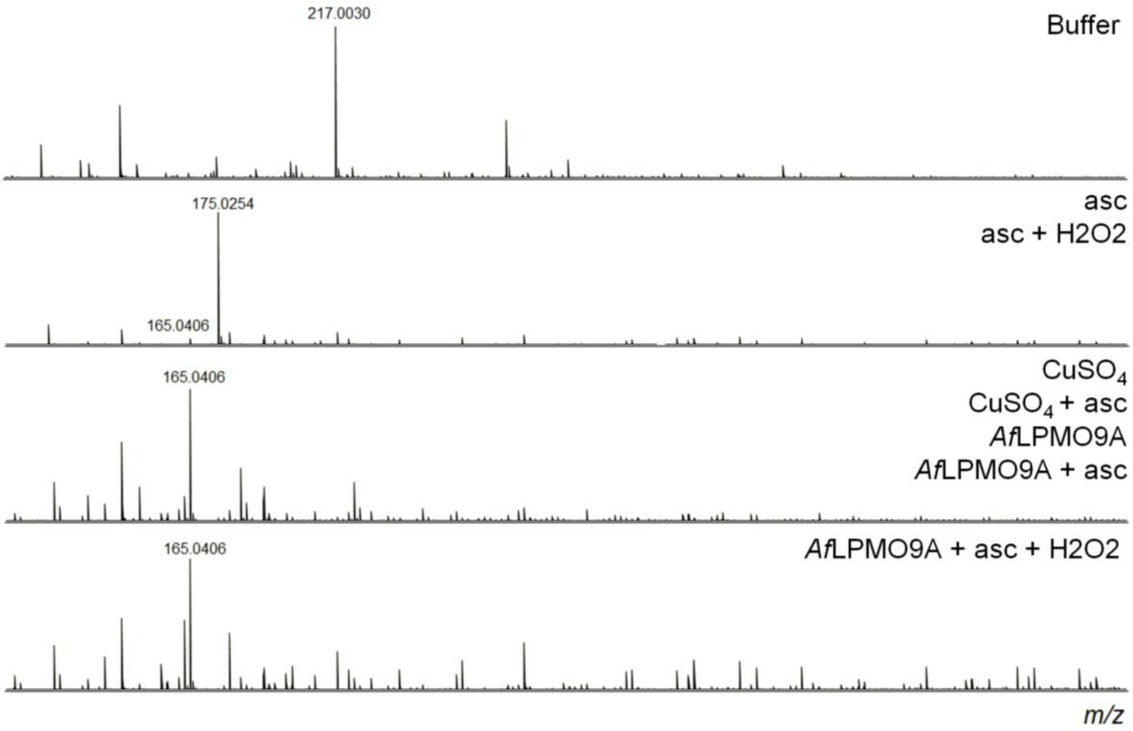

Figure S4. ESI-MS data. Reactions with milled PET bottle treated with buffer, ascorbate, ascorbate + H_2O_2, $CuSO_4$, $CuSO_4$ + ascorbate, *Af*LPMO9A, *Af*LPMO9A + ascorbate, and *Af*LPMO9A + ascorbate + H_2O_2. The controls and reactions were incubated in 0.02 M ammonium acetate buffer, pH 6.0, for 48h/37 ^{o}C/800 rpm. The intensity (%) of the peaks was calibrated taking the higher peak of each reaction into consideration. *m/z* 175.0254 corresponds to ascorbate M^{-1}. asc, ascorbate.

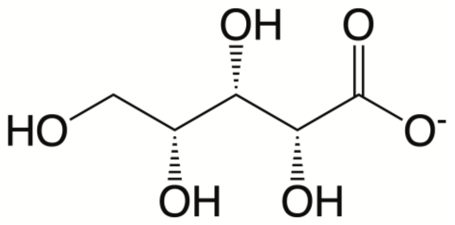

Figure S5. D-xylonate. D-xylonate is suggested as the soluble product released in reactions with PET as substrate ($C_5H_9O_6$, M-1=165.0406). It is an intermediate in the biosynthesis of EG from renewable sources[4].

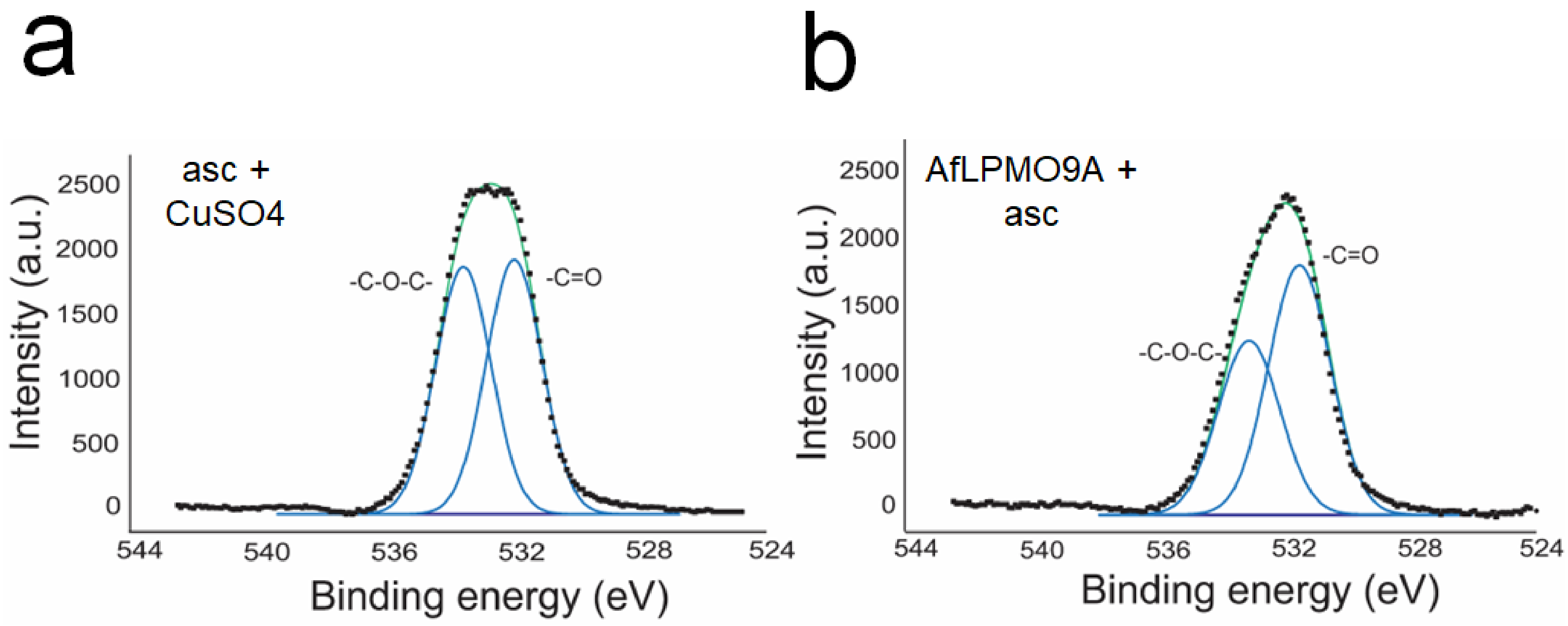

Figure S6. O(1*s*) XPS spectra. Ascorbate + $CuSO_4$ (control) (a), and *Af*LPMO9A + ascorbate-treated PET bottle (b). C=O, 531.5 eV; C-O-C, 533.1 eV. The O(1*s*) spectrum of PET bottle coupons treated with *Af*LPMO9A + ascorbate shows the emergence of C=O groups compared to the control. asc, ascorbate.

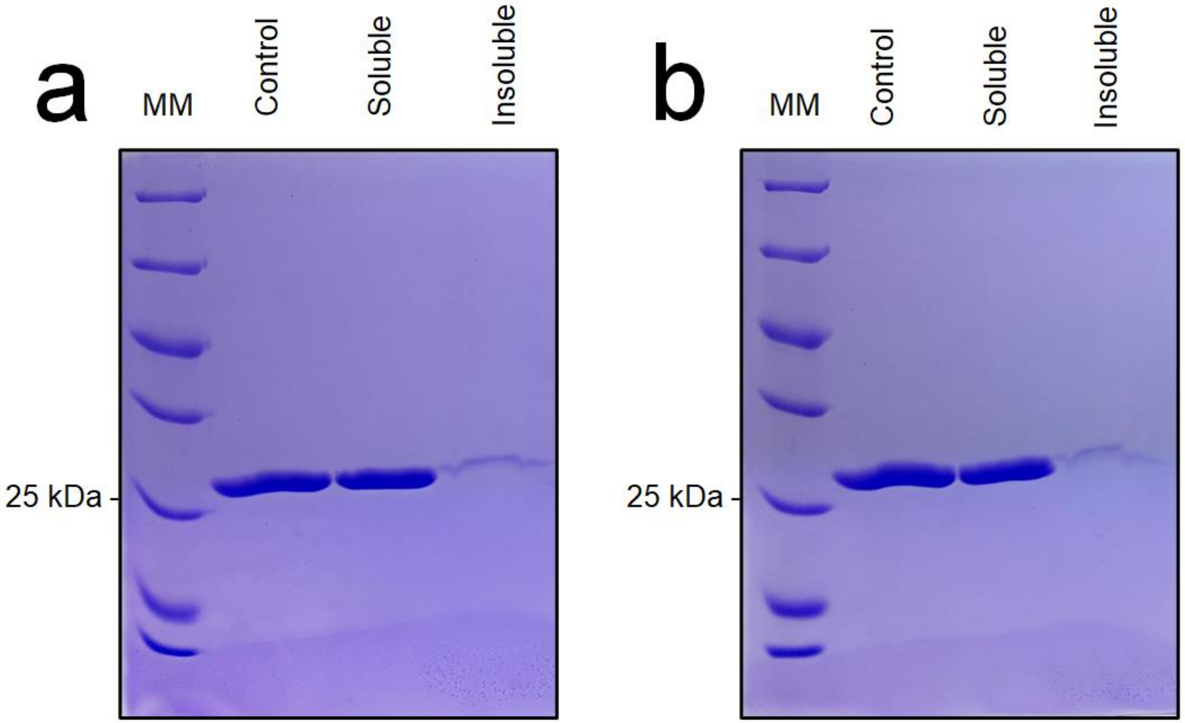

Figure S7. Binding of *Af*LPMO9A to PET coupon (a) and HDPE coupon (b). *Af*LPMO9A was incubated with PET or HDPE (0.05 M sodium phosphate buffer, pH 6.0) on ice. The SDS-PAGE binding assays were carried out as described in[5]. Control, *Af*LPMO9A incubated without substrate; soluble, *Af*LPMO9A in the soluble fraction (supernatant); insoluble, *Af*LPMO9A in the insoluble fraction (substrate). MM, molecular marker

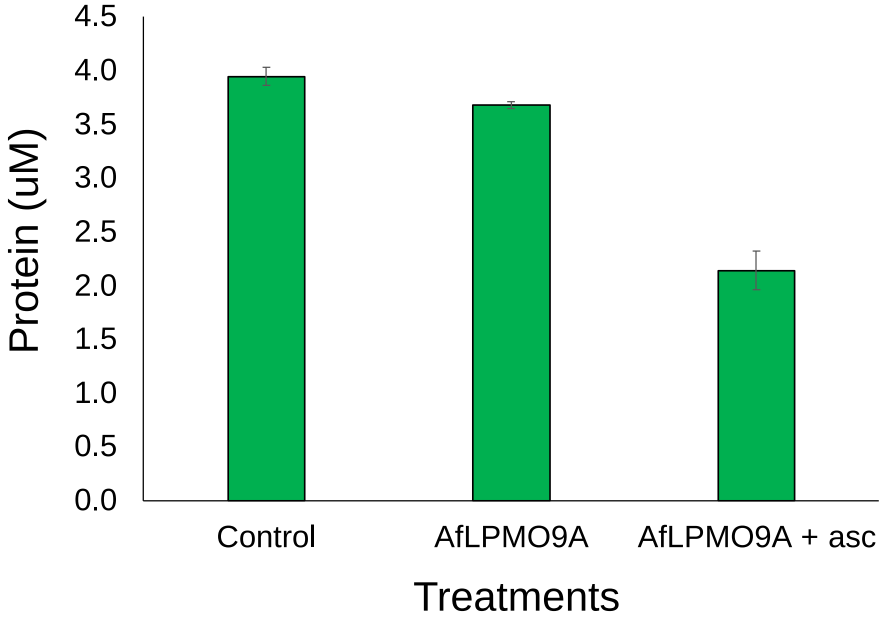

Figure S8. Binding of *Af*LPMO9A to milled PET. Milled PET was incubated with 4µM *Af*LPMO9A in 0.05 M sodium phosphate buffer, pH 6.0, at 37 ºC/850 rpm/48h. After incubation, the soluble fraction (supernatant) was read at 280 nm in spectrophotomer. Control, *Af*LPMO9A in the absence of substrate. Reactions carried out with PET and no *Af*LPMO9A were used as blank.

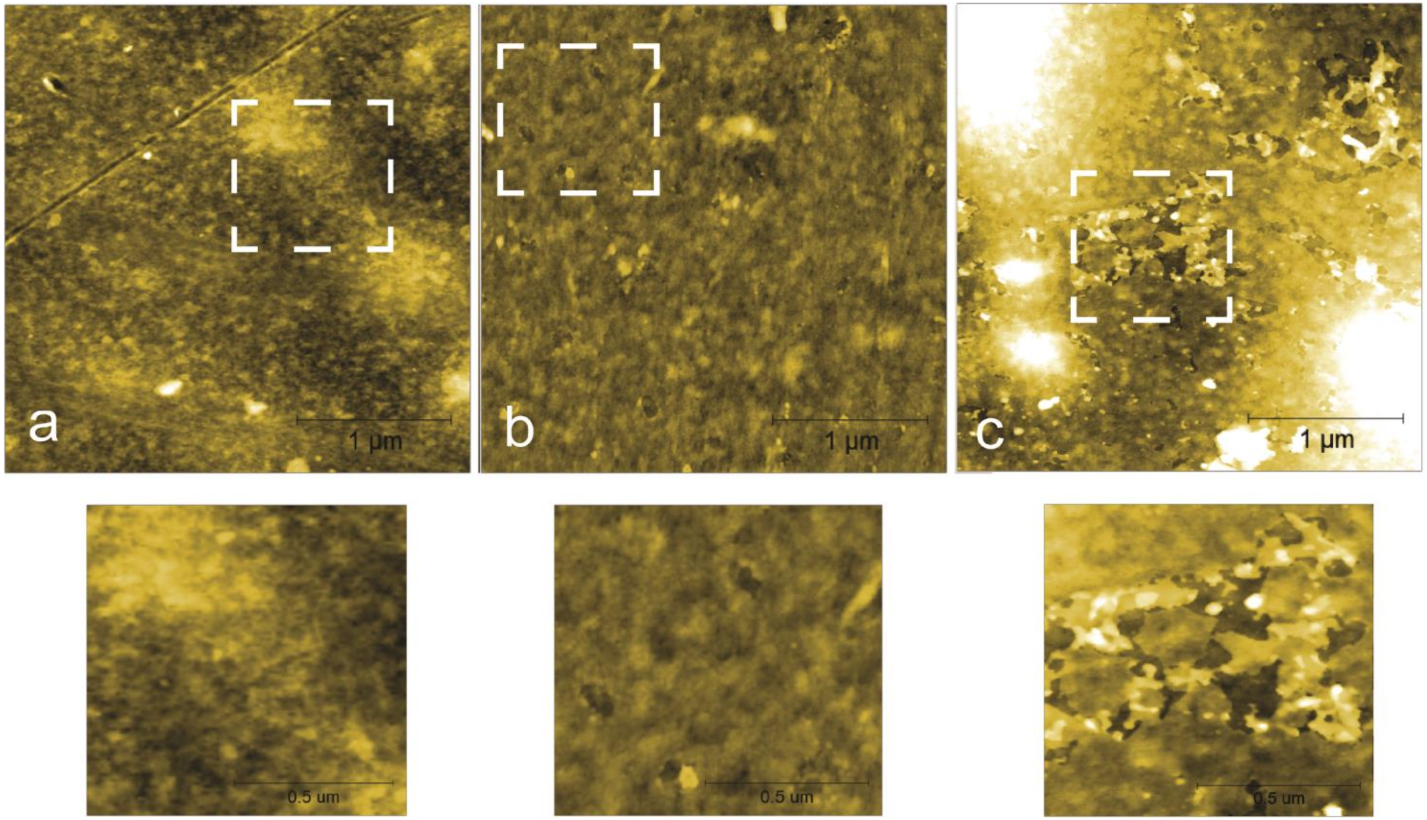

Figure S9. Topographies (3µm x 3µm) of *Af*LPMO9A-treated PET film (Mylar®). The PET film was incubated with buffer (a), *Af*LPMO9A (b), or *Af*LPMO9A +asc (c) in 0.05 M sodium phosphate buffer, pH 6.0, for 48 h at 37 ^{o}C/850 rpm followed by sonication before visualization by AFM. The 1µm x 1µm topographies correspond to the areas highlighted in 3µm x 3µm.

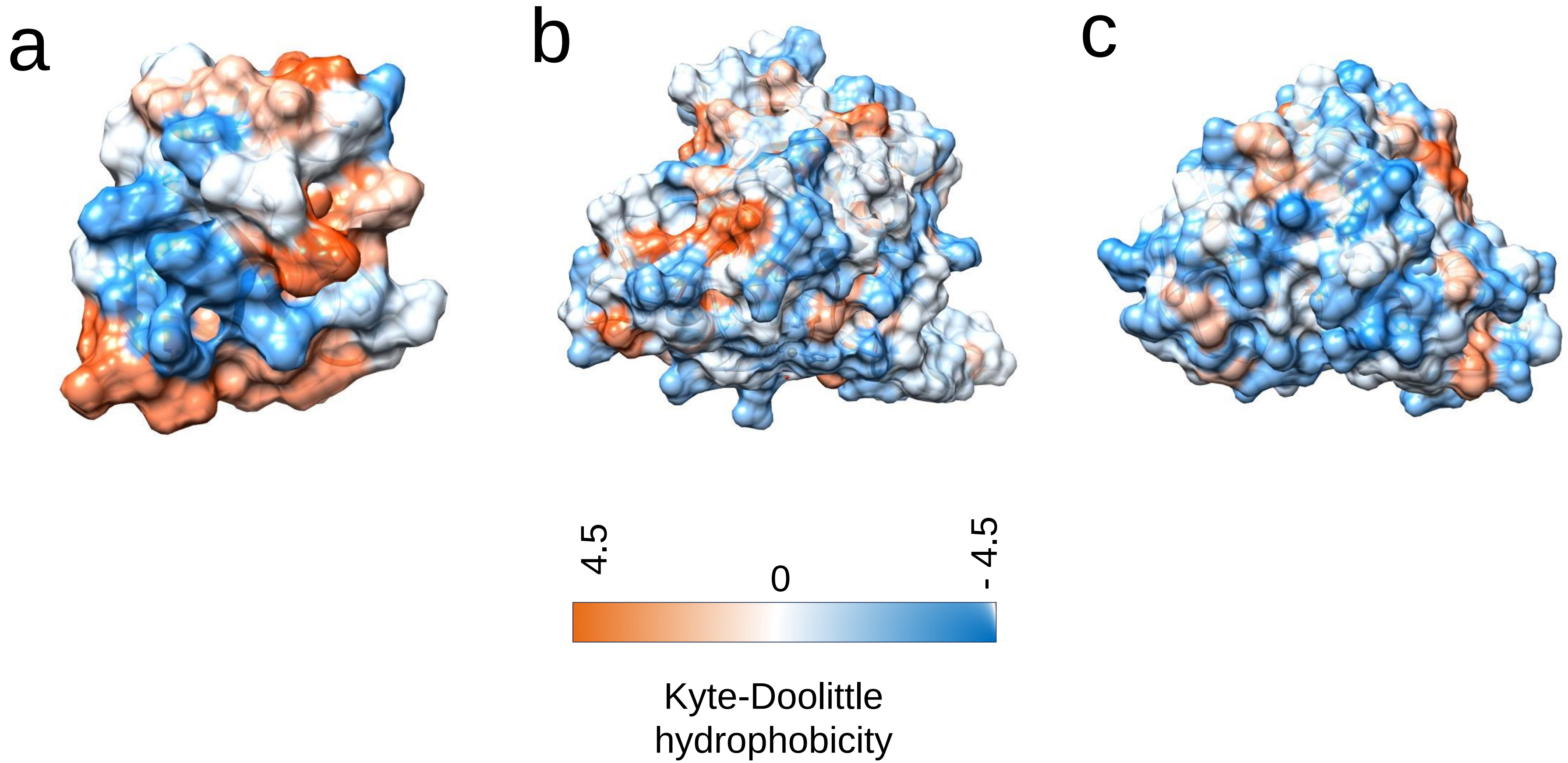

Figure S10. Surface of HFBII (hydrophobin) from *Trichoderma reesei* (PDB code: 2B97) (a), *Af*LPMO9A (7OVA) (b), and *Kp*LPMO10A (6NDQ) (c). The surfaces are coloured by hydrophobicity (Kyte-Doolittle scale) highlighting the hydrophobic patch displayed by HFBII, and the abundance of hydrophilic residues in the *Af*LPMO9A and *Kp*LPMO10A.

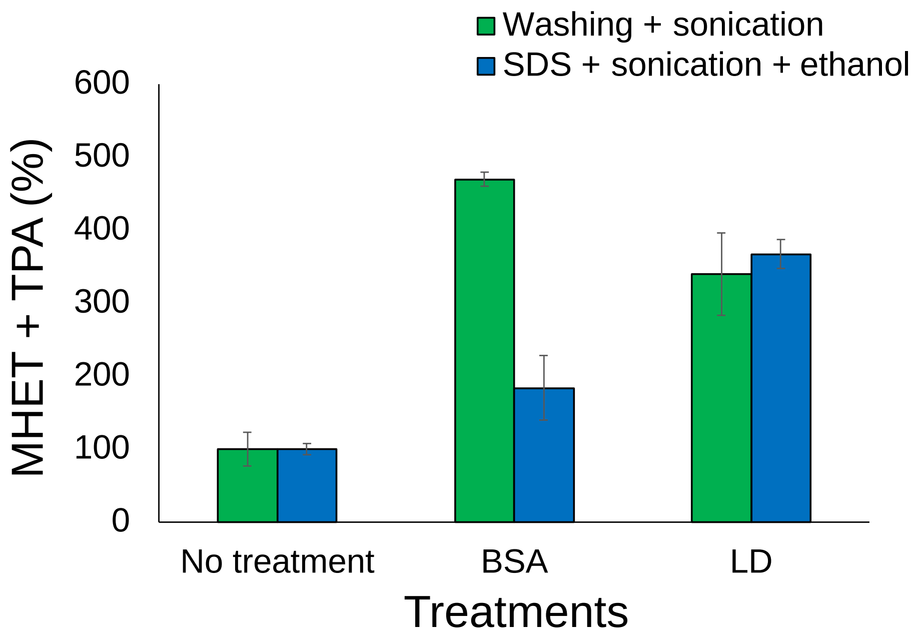

Figure S11. MHET and TPA detected in reactions with the sequential addition of BSA or LD and PETase to PET. The pre-treatment of milled PET bottle with 4μM BSA or LD were carried out in 0.05 M sodium phosphate buffer, pH 6.0, at 37 °C for 48 h/850 rpm. The residual substrate was washed by three steps of sonication in MilliQ water (green) or with SDS + sonication + ethanol (blue), followed by the addition of *Is*PETase (0.05 M sodium phosphate buffer, pH 7.2, at 30 °C for 120 h/400 rpm). The concentration of *Is*PETase used was 0.05 μM.

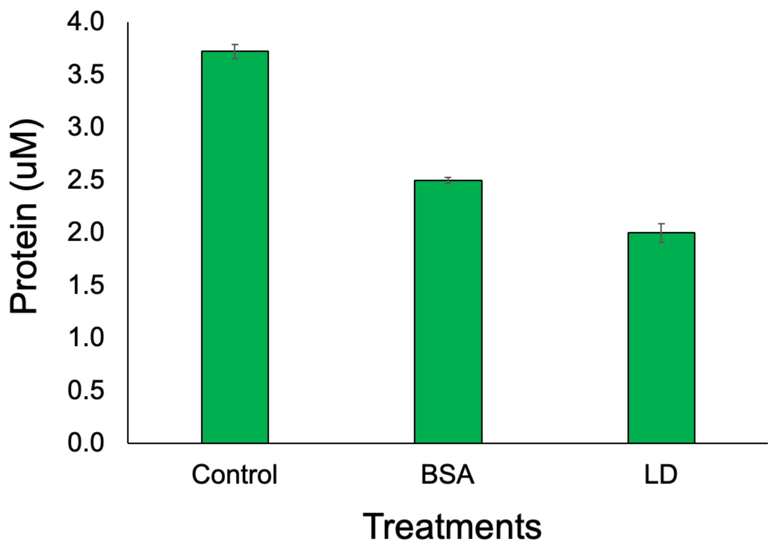

Figure S12. Binding of BSA and LD to milled PET. Milled PET was incubated with 4µM BSA or LD in 0.05 M sodium phosphate buffer, pH 6.0, at 37 ^{o}C/850 rpm/48h. After incubation, the soluble fraction (supernatant) was read at 280 nm in spectrophotomer. Control, the respective enzyme in the absence of substrate. Reactions carried out with PET and no enzyme were used as blank. BSA, bovine serum albumin; LD, lactate dehydrogenase.

Table S1. Calculated hydropathicity for proteins used in this work and hydrophobins from *Trichoderma reesei*

Proteins	GRAVY index
HFBI	0.269
HFBII	0.877
*Af*LPMO9A	-0.231
Bovine serum albumin	-0.479
Lactate dehydrogenase	0.044

The hydropathicity is a measurement of the hydrophobicity of a peptide. It calculated as the GRAVY index score using the ExPASy-ProtParam Tool (https ://web.expasy.org/protparam/). Hydrophobic and hydrophilic proteins have positive and negative GRAVY index, respectively.

Supporting Information – References

[1] D. Cannella, K.B. Möllers, N.-U. Frigaard, P.E. Jensen, M.J. Bjerrum, K.S. Johansen, C. Felby, *Nat. Commun*., 2016, **7**, 11134

[2] T.L.R. Corrêa, A.T. Junior, L.D. Wolf, M.S. Buckeridge, L.V. Santos, M.T. Murakami, *Biotechnol. Biofuels,* 2019, **12**, 117.

[3] C.L. Markert, R.L. Hunter, *J. Histochem. Cytochem.*, 1959, **7,** 42-49.

[4] P. Lomwongsopon, C. Varrone, *Fermentation*, **2022**, *8*, 47.

[5] L.I. Crouch, A. Labourel, P.H. Walton, G.J. Davies, H.J. Gilbert, *J. Biol. Chem.*, 2016, **291 (14)**, 7439-7449.
